# Retraction Note to: Long non-coding RNA LBX2-AS1 enhances glioma proliferation through downregulating microRNA-491-5p

**DOI:** 10.1186/s12935-020-01712-y

**Published:** 2020-12-28

**Authors:** Qunbang Chen, Jian Gao, Yingjia Zhao, Ruizhe Hou

**Affiliations:** 1 Department of Neurosurgery, China-Japan Union Hospital of Jilin University, Changchun, 130033 Jilin China; 2 Department of Gynecology and Obstetric, The Second Hospital of Jilin University, Changchun, 130000 Jilin China

## Retraction Note to: Cancer Cell Int (2020) 20:411 https://doi.org/10.1186/s12935-020-01433-2

The authors have retracted this article [1] because, after publication, they found errors in the calculation of the data on expression of LBX2-AS1 in glioma shown in Fig. 1C. The authors reviewed the data and found that there was no significant difference in LBX2-AS1 expression when comparing glioma tissue (Grades II, III and IV) with normal brains. Additionally, they did not find a clear trend in higher expression of LBX2-AS1 when comparing Grade III with Grade II or Grade IV with Grade III. Further work is currently being done to determine whether there is high expression of LBX2-AS1 in glioma and whether LBX2-AS1 is increased in high-grade glioma.

All authors agree to this retraction.
